# Personalized temporal interference stimulation targeting striatum reduces functional stability and dynamic connectivity variability in the sensorimotor network

**DOI:** 10.3389/fnins.2025.1645903

**Published:** 2025-09-26

**Authors:** Dongsheng Tang, Lang Qin, Longfei Hu, Siqi Gao, Yixuan Jian, Zhiqiang Zhu

**Affiliations:** School of Kinesiology, Shenzhen University, Shenzhen, China

**Keywords:** temporal interference stimulation, personalized brain stimulation, functional stability, dynamic functional connectivity, functional magnetic resonance imaging

## Abstract

**Background:**

Functional stability within brain networks, particularly the sensorimotor network (SMN), is crucial for coherent motor control. Temporal Interference (TI) stimulation offers a non-invasive method to modulate deep brain structures like the striatum, yet its impact on dynamic functional stability across motor networks remains largely unexplored.

**Methods:**

Twenty-six healthy male participants separately underwent TI stimulation and Sham stimulation in a crossover, double-blind, randomized controlled trial with counterbalanced protocol. resting-state functional magnetic resonance imaging (rs-fMRI) was acquired before and during the stimulation. A total of 20 min TI stimulation (10 mA, Δf = 20 Hz) was applied to the right striatum using personalized electrode montages optimized. Dynamic functional connectivity (dFC) was computed using a sliding-window approach. Voxel-wise functional stability across the whole brain was quantified by Kendall’s concordance coefficient of voxel-to-voxel dFC. Seed-based dFC variability in the right striatum was measured as the standard deviation of dFC across windows.

**Results:**

(1) Functional stability: TI stimulation significantly decreased functional stability in bilateral SMA regions (predominantly SMA proper, with parts of pre-SMA) compared to Sham and baseline conditions (*P* < 0.01). (2) Dynamic functional connectivity: TI stimulation reduced dFC variability between the right striatum and left SMA region (predominantly SMA proper, with parts of pre-SMA) compared to baseline (*P* < 0.01). (3) Safety: No adverse cognitive effects or side effects were observed, with good blinding effectiveness maintained throughout the study.

**Conclusion:**

Our findings indicate that TI stimulation targeting the striatum effectively modulates sensorimotor network stability and dFC variability within the cortico-striatal pathway, highlighting its potential as a non-invasive neuromodulation approach for motor network disorders.

**Clinical trial registration:**

[www.chictr.org.cn;], identifier [ChiCTR2500098699].

## 1 Introduction

The functional stability of the sensorimotor network (SMN) plays an indispensable role in ensuring accurate motor control and coordination during complex movements (Kong et al., 2021). As the central information hub in the brain that dominates motor control and sensory integration, the efficient operation of this network not only supports the smooth coordination of actions but also coordinates motor planning, execution, and rewards learning mechanisms through the striatum, a core node in the SMN (Bostan and Strick, 2018; Greene et al., 2020). The striatum achieves this by maintaining close connections with the cortex, thalamus, and other basal ganglia nuclei (Graybiel et al., 1994). A substantial body of evidence indicates that striatal dysfunction is closely associated with various neurodegenerative disorders, such as Parkinson’s disease (PD) and Huntington’s disease (HD) (Ring and Serra-Mestres, 2002). Moreover, the core symptoms of these diseases, such as bradykinesia, tremors, involuntary movements, and impulse control disorders, are directly linked to functional disturbances in the SMN (Ragothaman et al., 2023). Any abnormal fluctuations in the functional stability of the SMN can directly impact the accuracy and flexibility of motor functions, potentially leading to broader motor dysfunctions (Cai et al., 2018; Díez-Cirarda et al., 2018; Kim et al., 2017; Zhu et al., 2020). Therefore, precise neuro-modulation of the striatum holds promise for fundamentally improving the symptomatic manifestations of these diseases.

Neuro-modulation technologies such as deep brain stimulation (DBS), transcranial electrical stimulation (tES), and transcranial magnetic stimulation (TMS) have been developed and applied in clinical settings over the past few decades, offering new treatment options for patients with refractory motor and psychiatric disorders (Camacho-Conde et al., 2022; Krishna and Fasano, 2024). Each of these techniques has its unique advantages and applications in clinical practice. However, they still face significant limitations. Although the therapeutic efficacy of DBS has been demonstrated in numerous studies, it is inherently invasive and requires neurosurgical implantation and ongoing device management, with risks of hemorrhage, infection, hardware-related complications and stimulation-related adverse effects (Bronstein et al., 2011). In addition, most non-invasive stimulation modalities currently face significant challenges, including difficulty achieving both high spatial resolution and effective direct modulation of deep targets, particularly within core basal ganglia structures such as the striatum (Huang and Parra, 2019; Siebner et al., 2022), and insufficient modulation intensity, which constrains therapeutic efficacy (Wang et al., 2024; Wessel et al., 2023).

Based on this, the present study proposes and explores a novel neuro-modulation technology, Temporal Interference (TI) stimulation. TI utilizes the cross-interference of two slightly different high-frequency currents (such as 2 and 2.1 kHz) to generate low-frequency envelope waves (e.g., 100 Hz) in deep brain regions (e.g., thalamus and striatum), achieving deep neural modulation without the need for surgical electrode implantation (Grossman et al., 2017). TI stimulation overcomes the limitations of conventional non-invasive techniques in terms of targeting depth and stimulation intensity, while providing precise deep brain targeting capabilities comparable to DBS (e.g., hippocampus, basal ganglia) and maintaining the safety advantages of non-invasive approaches (Vassiliadis et al., 2024b). Additionally, previous studies have confirmed that it has the potential to modulate key neural circuits, such as the cortico-basal ganglia-thalamus circuit (Lamoš et al., 2025; Modak et al., 2024; Yang et al., 2024a,b). Based on this mechanism, a clinical trial for Parkinson’s disease tremor intervention has observed symptom improvement, but the efficacy varies among individuals (Yang et al., 2024b). However, the dynamic function of TI targeting the striatum on the SMN is still unclear.

Therefore, this study will systematically evaluate the precise localization and regulation capabilities of TI on the striatum using healthy subjects. Furthermore, it will examine the enhancing effects of TI on the functional stability of the sensorimotor network. Based on the objectives of this study, the following research hypotheses are proposed: First, TI can effectively regulate the striatum; second, TI stimulation of the striatum can effectively modulate the dynamic functional characteristics of the SMN.

## 2 Methods

### 2.1 Participants

A total of 28 healthy male participants completed the study. Two participants were excluded due to excessive head movement, leaving 26 participants for final analysis (mean age = 20.15 ± 1.51 years). All participants were right-handed (mean laterality quotient 86 ± 16.7) and assessed using the Edinburgh Handedness Inventory. Exclusion criteria included any history of neurological disorders, current medication use, metal implants, and previous adverse reactions to non-invasive brain stimulation (Wessel et al., 2023). The study was conducted at Shenzhen University and approved by the Medical Ethics Committee of Shenzhen University Health Science Center (project number 202400151) and pre-registered on the Chinese Clinical Trial Registry (www.chictr.org.cn; identifier: ChiCTR2500098699). Written informed consent was obtained.

### 2.2 Study design

**FIGURE 1:**
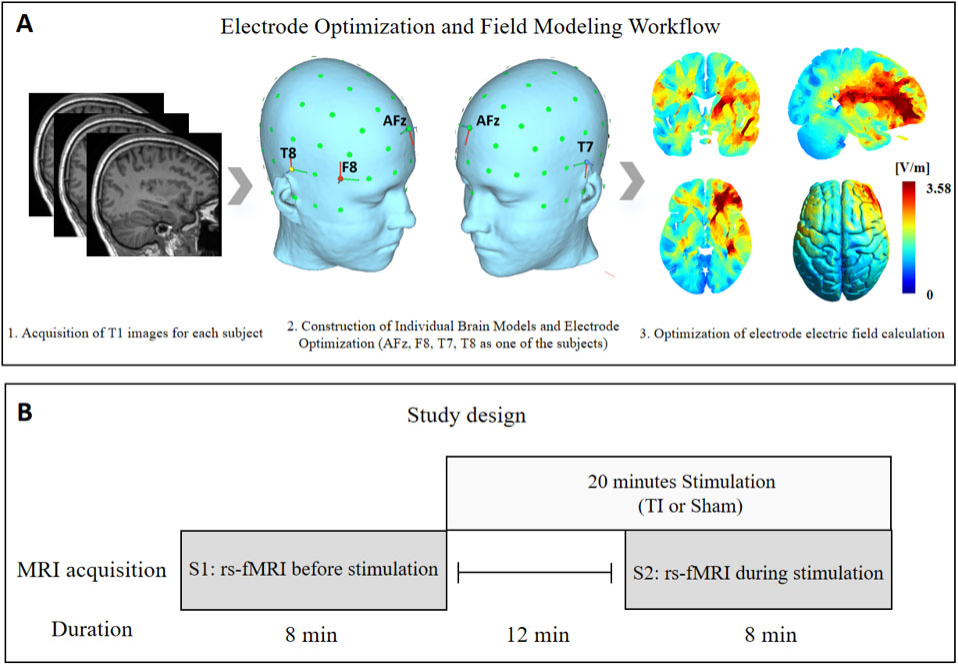
Study design. **(A)** Workflow for personalized stimulation electrode optimization and the concept map of electric field modeling. **(B)** Procedure for Magnetic Resonance Imaging (MRI) data acquisition during stimulation intervention. TI, temporal interference stimulation; rs-fMRI, resting-state functional magnetic resonance imaging.

This randomized, double-blind, crossover trial involved two groups: the TI group (TI stimulation) and the Sham group (sham stimulation). All participants were required to attend three experimental visits. During the first visit, high-resolution T1-weighted magnetic resonance imaging (MRI) scans were acquired to optimize the personalized stimulation electrode protocol (Figure 1A). In the second and third visits, participants were randomly assigned to receive either TI stimulation or sham stimulation, and resting-state functional MRI data were collected before stimulation (S1) and during stimulation (S2) (Figure 1B).

Regarding assessment scales, participants were required to complete the Stanford Sleepiness Scale (SSS) before each of the latter two visits to ensure a consistent state of wakefulness during scanning. Additionally, the Montreal Cognitive Assessment (MoCA) was administered before and after each session to evaluate any cognitive effects of the stimulation on the brain. Following the stimulation, participants filled out the Adverse Effects Questionnaire (AEQ) and underwent a blinding check to assess the effectiveness of blinding.

### 2.3 Stimulation parameter and personalized stimulus montage protocol

**FIGURE 2:**
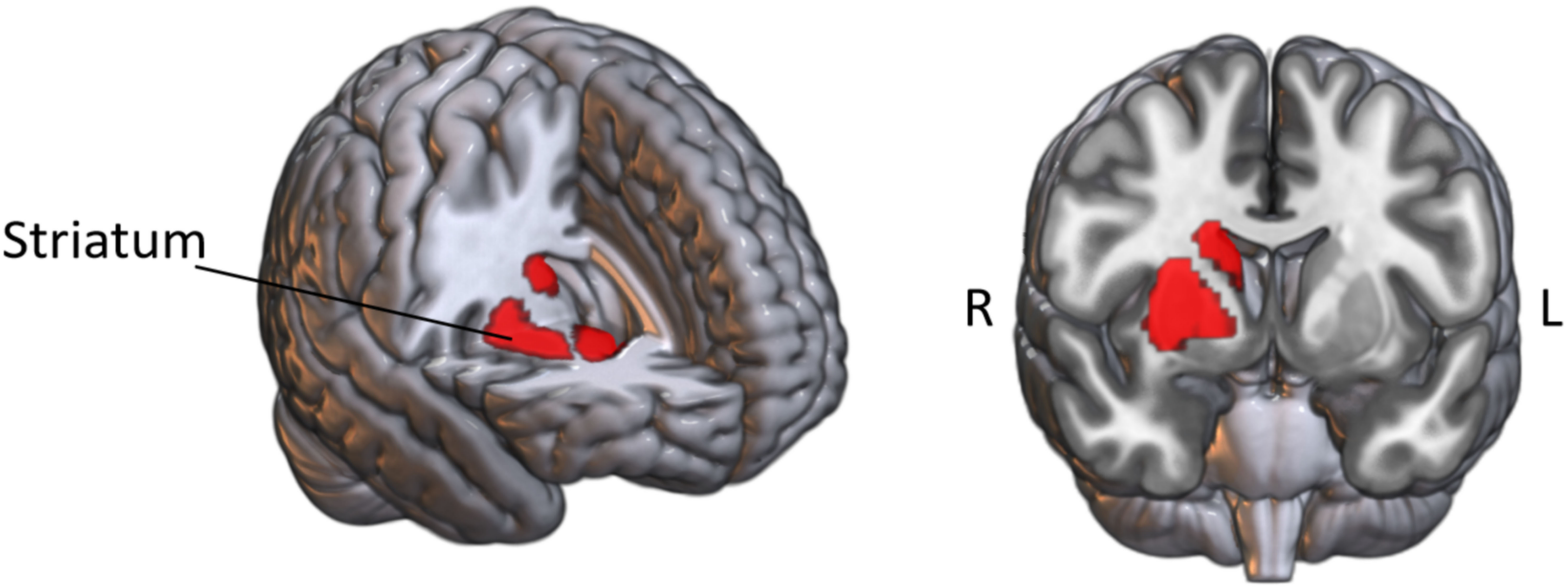
Stimulation targets. R, right; L, left.

The intervention was conducted by the NervioX-1000 neuromodulation system (Suzhou Brain Dome Technology Co., Ltd., Suzhou, China). The stimulation region-of-interest (ROI) was located in the right striatum (Figure 2). Based on the results of individualized electrode position optimization, four circular conductive rubber electrodes with a diameter of 2 cm were precisely placed using the 10-10 international standard EEG cap. Prior to electrode placement, abrasive gel was applied to remove skin keratin, and conductive gel was evenly applied between the scalp and the electrodes to ensure tight electrode-skin contact and reduce impedance. Two channels of high-frequency alternating current were applied (I_1_: 2 kHz and I_2_: 2.02 kHz), generating a low-frequency interference modulation of 20 Hz targeting the ROI, as this frequency is critical for motor control and aligns with findings in previous TI studies (Modak et al., 2024; Vassiliadis et al., 2024a; Wessel et al., 2023; Zhu et al., 2025). The peak-to-peak amplitude of the current was set at 10 mA (5 mA per channel), which is consistent with established safety parameters, as prior research has shown that currents up to 15 mA are safe and effective (Wang et al., 2024). The total stimulation duration of 20 min is a standard duration that has been validated in TI literature to enhance motor network connectivity (Yang et al., 2024b; Zhu et al., 2024). TI stimulation was administered only during the scanning session (not prior to scanning), with current maintained continuously throughout the stimulation period, including a 30 s ramp-up phase at initiation and a 30 s ramp-down phase at termination to ensure participant safety and comfort. Sham stimulation delivered current only during these 30 s ramp-up and ramp-down phases, with no current applied during the intermediate stimulation period. The impedance was kept below 15 kΩ during stimulation.

The electrode locations were optimized for each participant. This was done using the SimNIBS software to create a finite element model (FEM) of the brain from the structural images of the subject (Saturnino et al., 2018). Specifically, we segmented tissues and assigned conductivities, placed electrodes following the standard 10-10 EEG system of 64 channels, generated tetrahedral head meshes via Gmsh, performed FEM, and then calculated the electric field. The right striatum was targeted at MNI coordinates (28, 4, −4) from Wessel et al. (2023), using a 10 mm spherical ROI to optimize electric field intensity. This approach achieved an average electric field intensity of 2.92 V/m in the target region (Supplementary Figure 1), ensuring precise neuromodulation (for detailed electrode placement and electromagnetic computation data, see Supplementary Section A).

### 2.4 Image acquisition

Imaging data were collected using a Siemens Prisma 3.0-Tesla system (Erlangen, Germany) with a 64-channel head coil. High-resolution T1-weighted anatomical images were acquired using a 3D MPRAGE (magnetization-prepared rapid gradient echo) sequence: repetition time (TR) = 2,300 ms, echo time (TE) = 2.26 ms, flip angle (FA) = 8°, slice thickness = 1.6 mm, field of view (FOV) = 256 × 232 m^2^, voxel size = 1 × 1 × 1 mm^3^, total acquisition time (TA) = 8.92 min, 192 volumes. Rs-fMRI data were acquired using a gradient-echo echo-planar imaging (EPI) sequence (TR = 1,000 ms, TE = 30 ms, FA = 66°, slice thickness = 3 mm, FOV = 210 × 210 mm^2^, voxel size = 3 × 3 × 3 mm^3^, TA = 8.32 min, 488 volumes). Participants wore earplugs for noise protection and were instructed to remain awake, still, and focused on a fixation cross with open eyes, avoiding directed thoughts during the scanning sessions. To ensure a consistent state during the MRI scanning procedure, participants completed the Stanford Sleepiness Scale (SSS) (see Supplementary Figure 2 for results).

### 2.5 Data preprocessing

All preprocessing was performed using DPARSF V8.0 toolboxes (Yan et al., 2016). The first 8 volumes (8s) were removed to allow data to reach equilibrium, leaving a total of 480 volumes for final analysis. Images then underwent slice timing, head motion correction. Nuisance covariates, including linear trend, Friston et al. (1996) 24 head motion parameters, white matter signal, and cerebrospinal fluid signal, were regressed out from the functional signal. Then the functional images were normalized to Montreal Neurological Institute (MNI) space by Diffeomorphic Anatomical Registration Through Exponentiated Lie algebra (DARTEL) (Ashburner, 2007). Band-pass temporal filter (0.01–0.1 Hz) was applied to the normalized functional images.

To mitigate head motion effects, volume-based frame-wise displacement (FD) was calculated (Power et al., 2012). Timepoints with FD > 0.2 mm were marked as problematic and included as separate regressors during nuisance covariate regression (Yan et al., 2013). Following common practice in neuroimaging, we applied a head motion control criterion excluding participants whose mean FD exceeded three standard deviations (SD) from the sample mean to minimize outlier influence(España-Irla et al., 2025; Yan et al., 2013). Finally, two participants were excluded under the head motion control criteria, and 26 participants were included in the subsequent analysis.

### 2.6 Calculation of functional stability

According to recently published studies (Li et al., 2020; Zhu et al., 2020), functional stability for a brain voxel was defined as the concordance of its voxel-level dynamic functional connectivity (dFC) over time within a scanning session. The functional stability characteristics were calculated using the Stability Analysis toolkits in DPABI software, employing a sliding-window approach with a window size of 64 TR (64 s) and a sliding step of 4 TR (4 s) (Hutchison et al., 2013; Li et al., 2020; Zhu et al., 2020). Analyses were conducted in a voxel-by-voxel approach. For each voxel, Pearson’s correlation coefficients were calculated between its time course and those of all other voxels within the gray matter mask, resulting in a series of dFC maps across time windows for that voxel. Then, functional stability of that voxel was quantified by using Kendall’s concordance coefficient (KCC) of these dFC maps with time windows as raters based on the following equation:


$$K\,C\,C\,\frac{\sum_{n=1}^{N}R_{n}^{2}-\frac{1}{N}\,{\left(\sum_{n=1}^{N}R_{n}\right)}^{2}}{\frac{1}{12}\,K^{2}\,\left(N^{3}-N\right)}$$

where K is the number of windows, N is the number of connections of that voxel with all voxels within the gray matter mask, and Rn is the sum of rank for the n-th connection across all windows. The gray matter mask used to confine analyses in this study was created by thresholding the mean gray matter density map across participants at 0.2 and intersected with a group-level mask of 90% coverage of all functional images. For each window, connections are ranked across all voxels based on their functional connectivity strength. After obtaining the ranks for each connection within every window, the ranks for each connection are summed across all windows, yielding the Rn values for each connection. These summed ranks are then used in the KCC formula to quantify the temporal stability or consistency of the connection ranks. Specifically, KCC is calculated for each voxel as shown above, where a higher KCC value indicates greater concordance (i.e., stability) of that voxel’s dFC rankings across the different time windows. After obtaining the functional stability maps, z-standardization was performed within the gray matter mask, followed by spatial smoothing using a 8 mm full-width at half-maximum (FWHM) Gaussian kernel. Because our study primarily targets the SMN, we defined the network-level functional stability of the SMN for each participant as the spatial mean of voxel-wise stability values computed exclusively within the SMN mask.

### 2.7 Calculation of seed-based dFC variability

Seed-based dFC variability was estimated using a spherical ROI with a 5 mm radius, a commonly adopted size that balances spatial specificity and noise in connectivity analyses (Liu, 2011) and has been used in TI stimulation connectivity studies (Zhu et al., 2024, 2025). The ROI was centered at MNI coordinates (28, 4, −4), corresponding to our right striatum stimulation target and the site of maximal electric field intensity in our finite element modeling simulations. The dFC variability characteristics of the right striatum was calculated using the Temporal Dynamic Analysis (TDA) toolkits in DPABI software. The Hamming sliding window was selected for the whole-brain blood oxygenation level dependent signal time series. A window length of 100 TR (100 s) and a step width of 3 TR (3 s) were selected for dFC analysis (Yan et al., 2017). Previous studies have suggested that to exclude spurious fluctuations, the selected window length satisfied the criterion of being larger than 1/f min (1/0.01 s = 100 s), where f min represents the minimum frequency of the time series (Chen et al., 2022; Leonardi and Van De Ville, 2015; Li et al., 2019). In total 127 sliding windows of dFC were obtained. For each sliding window, Pearson’s correlation maps were produced by computing the temporal correlation coefficient between the truncated time series of the seed region and each voxel in the whole-brain gray matter mask. To improve the normality of the correlation distribution, each correlation map was first converted into a z-value map using Fisher’s r-to-z transformation. The dFC variability was then quantified as the SD of the 127 sliding-window z-value maps, followed by z-standardization of the resulting dFC maps.

### 2.8 Statistical analysis

**FIGURE 3:**
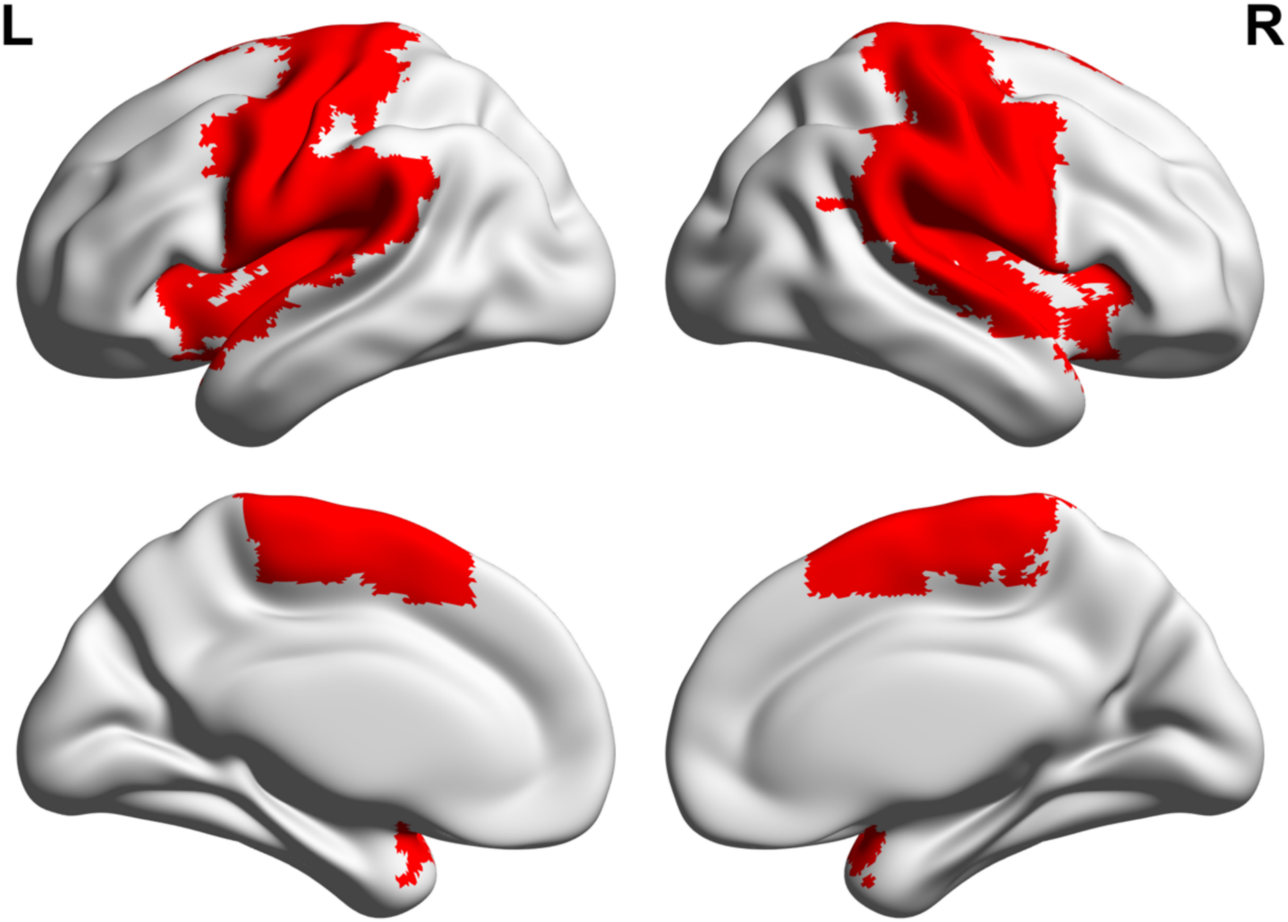
Distribution of sensorimotor network regions. R, right; L, left.

Functional MRI time series were analyzed using the DPABI (Data Processing and Analysis for Brain Imaging) software, specifically version 8.0, within MATLAB 2023a. A two-way repeated measures ANOVA was performed with two factors: group (two levels: TI and Sham) and time (two levels: pre-stimulation and during-stimulation). The dependent variables in this analysis included the effects of functional stability and the variability in seed-based dFC. Since the primary focus is on the SMN, the SMN network regions were used as masks for statistical analysis (Figure 3). According to Suo et al. (2022), the SMN network was parcellated using the automated anatomical labeling (AAL) atlas, which defines 20 regions including the bilateral precentral and postcentral gyri, supplementary motor area, Rolandic operculum, paracentral lobule, insula, supramarginal gyrus, superior temporal gyrus, Heschl’s gyrus, and temporal pole (superior temporal gyrus). Gaussian Random Field (GRF) correction was applied, with statistical thresholds set at voxel-level *p* < 0.005 and cluster-level *p* < 0.05. For brain regions exhibiting interaction effects, the corresponding activation values were extracted. Post-hoc tests were conducted using SPSS v26.0. To control for type I error inflation due to multiple comparisons, the significance level (α) in the correlation analysis was adjusted to 0.00625 (0.05/8) using the Bonferroni correction.

Pearson’s chi-square test was used to assess blinding efficacy, the Stanford Sleepiness Scale (SSS) and Adverse Effects Questionnaire (AEQ). For the non-normally distributed data of the Montreal Cognitive Assessment (MoCA), Generalized Estimating Equations (GEE) were applied to evaluate the interaction effects of group (TI and Sham) × time (pre-stimulation and post-stimulation). Statistical significance was set at *p* < 0.05.

## 3 Results

### 3.1 Data normality analysis

The normality of the four groups of data analyzed for the calculation of functional stability and seed-based dFC variability is as follows. For the TI-S1 group in functional stability, the Shapiro-Wilk test indicated compliance with a normal distribution (W = 0.944, *p* = 0.165), while the TI-S2 group also showed normal distribution characteristics (W = 0.944, *p* = 0.170). In the Sham-S1 group, normality was confirmed (W = 0.950, *p* = 0.229), and the Sham-S2 group exhibited a strong adherence to normality (W = 0.981, *p* = 0.888) (Supplementary Figure 5A).

Regarding seed-based dFC variability, the TI-S1 group showed normality (W = 0.937, *p* = 0.116) and the TI-S2 group was close to significance (W = 0.923, *p* = 0.052). The Sham-S1 group confirmed normality (W = 0.984, *p* = 0.947), and the Sham-S2 group also indicated strong adherence to normal distribution (W = 0.985, *p* = 0.959) (Supplementary Figure 5B). These findings suggest that all groups conform to normal distribution, which is crucial for subsequent statistical analyses.

### 3.2 The impact of functional stability analysis

**FIGURE 4:**
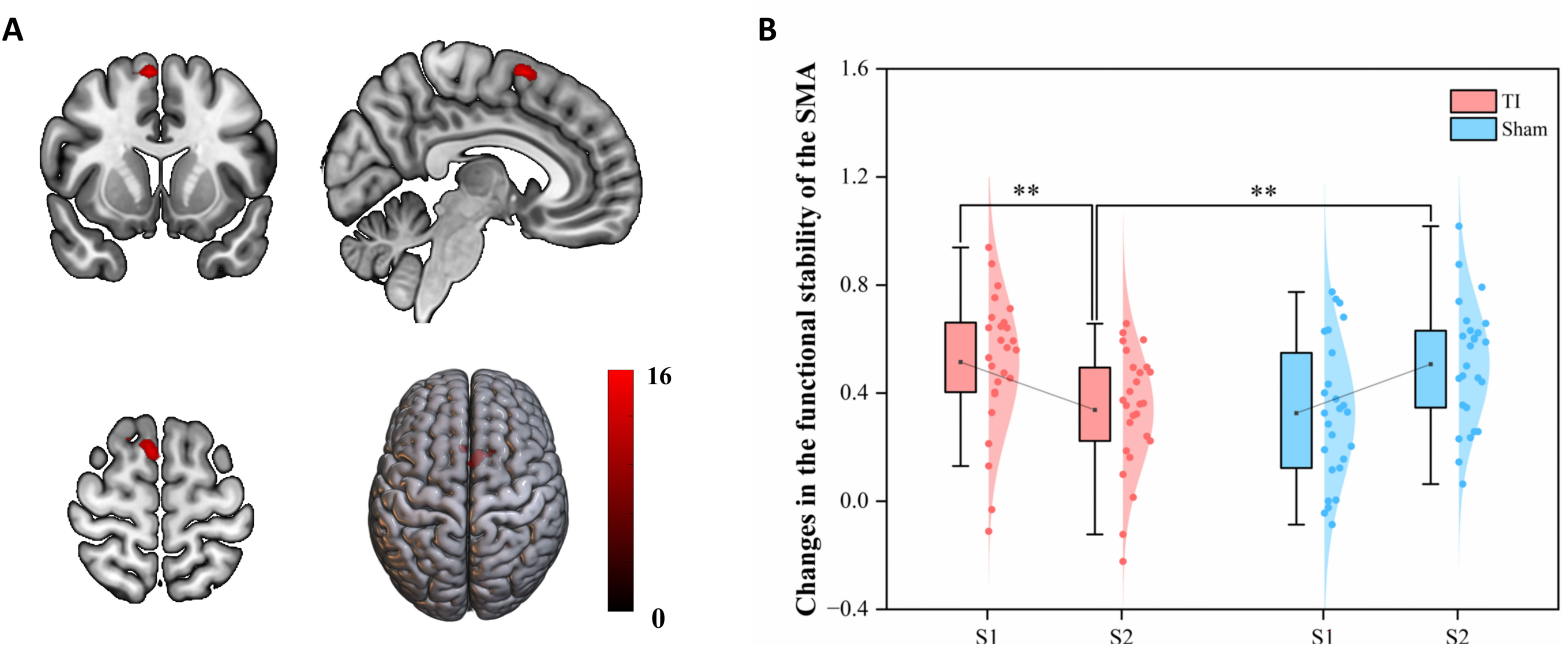
Effects of temporal interference (TI) stimulation on the functional stability. **(A)** Brain regions showing significant interaction effects. **(B)** *Post hoc* analysis of the functional stability metric (KCC) in the significant region-of-interests (ROIs0. TI, temporal interference stimulation; SMA, supplementary motor area; KCC, Kendall’s concordance coefficient; **P < 0.01.

Two-way repeated measures ANOVA results revealed significant interaction effects in left SMA (MNI: −6, 18, 54) and right SMA (MNI: 9, 12, 63) (GRF corrected, voxel-wise *P* < 0.005, cluster-wise *P* < 0.05) (Figure 4 and Table 1). The left SMA region was predominantly located in SMA proper (91.67%) with minor extension into pre-SMA (8.33%), while the right SMA region was entirely within SMA proper (100%). Subsequently, the regions showing significant interaction effects were extracted as ROIs, and the functional stability metric for each individual ROI was analyzed. The further analysis indicates that the Group × Time interaction effect was significant with F(1, 25) = 18.951, *p* < 0.001, partial eta squared = 0.431. The main effect of Group was not significant [F(1, 25) = 0.047, *p* = 0.831, partial eta squared = 0.002], nor was the main effect of Time [F(1, 25) = 0.002, *p* = 0.964, partial eta squared = 0.001]. *Post hoc* comparisons revealed that

**TABLE 1:** Brain regions with significant interaction effects on functional stability.

Comparisons	Brain region(s) and anatomical distribution	Peak MNI coordinates			Cluster voxels	Peak Z values
		x	y	z		
Interaction effect: Group (TI and Sham) × time (S1 and S2)	Left SMA (91.67% SMA proper, 8.33% pre-SMA)	−6	18	54	6	12.049
	Right SMA (100% SMA proper)	6	12	63	24	15.156

Significant clusters are shown for Gaussian Random Field (GRF) correction applied at voxel-wise *P* < 0.005 and cluster-wise *P* < 0.05. Anatomical localizations were determined using the AAL3 template. SMA, supplementary motor area.

TI-S2 significantly decreased the functional stability of the SMN, particularly in the bilateral SMA (*P* < 0.01), compared to Sham-S2 and TI-S1. No differences were observed at baseline, and no significant differences were found in other comparisons. These findings indicate that TI stimulation of the striatum leads to a significant decrease in functional stability in the bilateral SMA.

### 3.3 The impact of seed-based dFC variability

**FIGURE 5:**
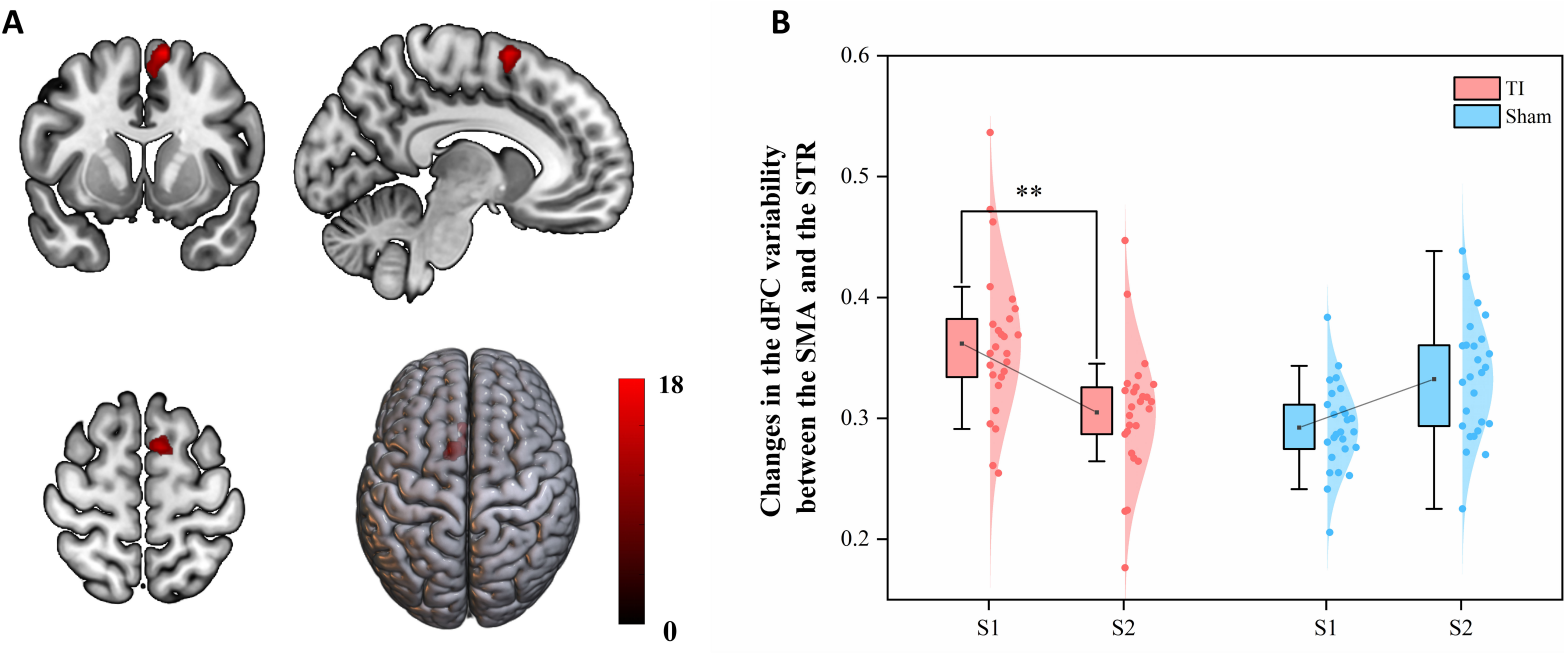
Effects of temporal interference (TI) stimulation on the dynamic functional connectivity (dFC) variability. **(A)** Brain regions showing significant interaction effects. **(B)** *Post hoc* analysis of the dFC variability metric (SD) in the significant region-of-interests (ROIs). TI, temporal interference stimulation; SMA, supplementary motor area; SD, standard deviation; **P < 0.01.

Two-way repeated measures ANOVA results revealed a significant interaction effect in the left SMA region (MNI: −12, 9, 66) (GRF corrected, voxel-wise *P* < 0.005, cluster-wise *P* < 0.05) (Figure 5 and Table 2). This region was predominantly located in SMA proper (96.15%) with minimal extension into pre-SMA (3.85%). By extracting brain regions with interaction effects as ROIs, the analysis showed that the Group × Time interaction effect was significant with F(1, 25) = 17.827, *p* < 0.001, partial eta squared = 0.416. The main effect of Group was significant [F(1, 25) = 4.409, *p* = 0.046, partial eta squared = 0.150], while the main effect of Time was not significant [F(1, 25) = 0.749, *p* = 0.395, partial eta squared = 0.029]. Post hoc tests indicated that TI-S2 significantly decreased the dFC variability between the right striatum and left SMA (*P* < 0.01) compared to the TI-S1 condition. No differences were observed at baseline, and no significant differences were found in other comparisons. These results suggest that TI stimulation significantly reduces the dFC variability between the striatum and left SMA, indicating an enhancement in the stability of functional connectivity between these two regions.

**TABLE 2:** Brain regions with significant interaction effects on dynamic functional connectivity (dFC) variability.

Comparisons	Brain region(s) and anatomical distribution	Peak MNI coordinates			Cluster voxels	Peak Z values
		x	y	z		
Interaction effect: group (TI and Sham) × time (S1 and S2)	Left SMA (SMA proper 96.15%, pre-SMA 3.85%)	−12	9	66	51	17.073

Significant clusters are shown for Gaussian Random Field (GRF) correction applied at voxel-wise *P* < 0.005 and cluster-wise *P* < 0.05. Anatomical localizations were determined using the AAL3 template. SMA, supplementary motor area.

### 3.4 Safety and blinding efficacy of stimulation parameters

The Stanford Sleepiness Scale (SSS) scores showed no significant differences between groups before experiment (χ^2^ = 4.275, *p* = 0.513) (Supplementary Figure 2A). Post-experiment blinded test revealed no differences in stimulation perception across groups (χ^2^ = 1.711, *p* = 0.425) (Supplementary Figure 4). The Montreal Cognitive Assessment (MoCA) scores showed no significant group × time interaction (*p* = 0.255) (Supplementary Figure 2B). The Adverse Effects Questionnaire (AEQ) indicated no significant differences in adverse effects between groups (χ^2^ = 10.864, *p* = 0.285) (Supplementary Figure 3). These findings demonstrate the safety and effective blinding of the stimulation protocol.

## 4 Discussion

In the present study, we found that TI stimulation induced significant alterations in both functional stability and dFC variability within the SMN. Specifically, TI stimulation resulted in a marked decrease in the functional stability of bilateral SMA regions (predominantly SMA proper, with parts of pre-SMA). Moreover, TI stimulation significantly reduced dFC variability between stimulation target (striatum) and the left SMA region (predominantly SMA proper, with parts of pre-SMA), indicating enhanced stability of functional connectivity between these two regions. These findings demonstrate that TI stimulation targeted at striatum notably modulates both spatial and dynamic characteristics of SMN activity, particularly within SMA-related regions. Additionally, we found that a stimulation current of 10 mA (5 mA per channel) applied for 20 min resulted in effective blinding and was associated with no severe adverse effects, thereby supporting its feasibility for clinical applications.

In the current study, we found TI stimulation produced a marked reduction in the functional-stability metric of the SMN, with the strongest effect located in the bilateral SMA. For a voxel or region, a higher stability metric means that its dynamic functional architecture configuration is more consistent and stable over time, and a lower stability metric reflects its ability to frequently and rapidly shift from one brain state to another (Li et al., 2020; Zhu et al., 2020). Previous studies have consistently demonstrated that the SMN exhibits low stability across the majority of brain states, implying a constant need to reorganize its functional connections to accommodate external environmental fluctuations and top-down influences from higher-order cortical regions. Because these SMN regions accumulate information over comparatively short temporal windows, they are able—and indeed required—to “frequently and rapidly shift from one brain state to another,” a property that rests on their capacity for fast reconfiguration of connectivity (Lerner et al., 2011). Against this backdrop, the additional drop in SMN stability that we observed after TI stimulation of the striatum likely reflects an enhanced demand for flexibility within cortical sensorimotor circuits, possibly facilitating rapid updating of motor plans and integration of dynamic afferent input. Our results provide further explanation for previous research. Prior studies have demonstrated, by assessing static BOLD signal activation with fMRI, that TI stimulation targeting striatum can modulate the brain’s SMN and enhance motor skill learning performance (Wessel et al., 2023). The present functional stability analyses suggest that these behavioral improvements may rely, at least in part, on increased temporal flexibility of the SMA centered SMN.

Moreover, clinical neuroimaging studies have shown that patients with subcortical neurodegenerative disorders, such as PD, exhibit more highly integrated but fewer highly segregated brain states (Díez-Cirarda et al., 2018; Kim et al., 2017). The increased abnormal functional stability may limit the ability to rapidly transition from one brain state to another, and these changes are associated with the severity of motor symptoms (Díez-Cirarda et al., 2018; Kim et al., 2017). Owing to its proven ability to non-invasively and safely engage deep subcortical nuclei while finely adjusting the temporal stability of brain functional networks, TI stimulation constitutes a potent next-generation neuromodulation technique. These dual properties make TI a promising therapeutic avenue for diseases marked by subcortical dysfunction and disrupted network dynamics (Cassarà et al., 2025a,b; Vassiliadis et al., 2024a).

Our study also found that TI stimulation targeting the striatum reduced dFC variability between the SMA and striatum, implying a more stable cortico-striatal coupling after stimulation. Physiologically, the striatum serves as the main gateway through which cortical motor commands enter the basal ganglia. A tighter and less volatile SMA-striatal connection therefore suggests that cortical volleys reach medium spiny neurons with higher signal-to-noise ratio, providing a more reliable “gating” signal for downstream pallidal and thalamic nuclei (Cruz et al., 2023; Shipp, 2017). From a network-dynamics perspective, this finding complements the decreased regional stability we observed in the SMA itself. Together, they hint at a functional reallocation in which the deep relay node (striatum) becomes more rigid to secure information throughput, while the cortical executor (SMA) retains the flexibility needed to rapidly update motor plans (Li et al., 2020; Mueller et al., 2015; Shehzad et al., 2009; Tomasi et al., 2017). Such a division of labor is consistent with computational models positing that stable basal-ganglia loops facilitate the consolidation phase of motor learning, whereas flexible cortical circuits support exploration in early learning stages (Baladron et al., 2023). Clinically, deficient or overly synchronized cortico-striatal coupling is a hallmark of disorders such as PD and Huntington’s disease; the ability of TI to normalize this coupling without surgery therefore holds translational promise (Hnazaee and Litvak, 2023; Naze et al., 2018; Zold et al., 2012). Collectively, our findings show that TI can recalibrate brain functional networks stability and therefore holds significant potential as a non-invasive therapeutic modality for brain disorders marked by abnormal network dynamics.

## 5 Limitation

This study has several limitations that must be considered. First, the lack of corresponding behavioral experiments limits our understanding of how the changes in functional stability induced by TI stimulation translate into motor performance improvements. Second, our analysis focused solely on immediate stimulation effects, precluding an understanding of long-term neuroplastic changes that may occur as a result of TI stimulation. This limitation restricts our ability to ascertain whether the observed alterations in brain functional network dynamics are transient or if they lead to enduring modifications in brain network dynamics and motor function over time. Third, the all-male cohort, which constrains generalizability to females. Future studies need recruit female participants, and test sex-by-condition interactions to determine whether effects differ by sex. Fourth, although finite element modeling was utilized for personalized targeting of the striatum, the lack of further measurement of off-target effects limits our ability to fully evaluate the efficacy of TI stimulation. Future research should focus on comprehensive assessments of these off-target influences.

## 6 Conclusion

Our study demonstrates that TI stimulation targeting the striatum modulates the stability of the SMN and the variability in dFC within the cortico-striatal pathway. These results underscore the potential of TI stimulation as a promising non-invasive neuromodulation technique for addressing disorders characterized by disrupted motor network dynamics.

## Data Availability

The data that support the findings of this study are openly available at the following URL: https://data.mendeley.com/datasets/bvzgv94txn/1.
