# Supplementary material for: Personalized temporal interference stimulation targeting striatum reduces functional stability and dynamic connectivity variability in the sensorimotor network
Source: Front Neurosci. 2025 Sep 26;19:1645903. doi: 10.3389/fnins.2025.1645903 (PMC12511026; doi:10.3389/fnins.2025.1645903)
Supplement: Supplementary file 1 [file Data_Sheet_1.docx]

Supplementary Material

# Section A: Protocol for optimizing personalized TI stimulation electrode montage (Main article - Section 2.3 continued)

This study utilized SimNIBS to segment the T1-weighted MRI images of the participants (1). Dividing their heads into six tissue types and assigning corresponding conductivities: scalp (0.465 S/m), skull (0.01 S/m), cerebrospinal fluid (1.65 S/m), white matter (0.126 S/m), gray matter (0.276 S/m), and cavity (2.5e-14 S/m). After segmentation, we registered the 10-10 EEG electrode system onto the participants' scalp.

Next, using the Gmsh, we generated tetrahedral finite element meshes of the participants' heads. After obtaining the meshes, we employed the finite element solver to compute the electric field intensity in the participants' brains. The electric field intensity was calculated separately for each of the two electrode pairs. Let E1 represent the magnitude of the electric field at each element of the grid (head model) due to a 5-mA current passing through one pair of electrodes, and E2 represent the electric field magnitude for the other pair of electrodes. The maximum amplitude modulation of the interference pattern is given by the following equation (2):

$$\boldsymbol{TI}_{\boldsymbol{max}}\mathbf{=}\left\{ \begin{aligned} \mathbf{2}\boldsymbol{E}_{\mathbf{2}}\mathbf{,}\boldsymbol{if} \boldsymbol{E}_{\mathbf{2}}\mathbf{<}\boldsymbol{E}_{\mathbf{1}}\mathbf{cos(}\boldsymbol{a}\mathbf{)} \\ \frac{\mathbf{(2}\left| \vec{\boldsymbol{E}_{\mathbf{2}}}\boldsymbol{\times(}\vec{\boldsymbol{E}_{\mathbf{1}}}\mathbf{-}\vec{\boldsymbol{E}_{\mathbf{2}}}\mathbf{)} \right|\mathbf{)}}{\left| \vec{\boldsymbol{E}_{\mathbf{1}}}\mathbf{-}\vec{\boldsymbol{E}_{\mathbf{2}}} \right|}\mathbf{, &}\boldsymbol{otherwise} \end{aligned} \right.$$

Here, E2<E1 and a < 90 degrees, and note that the TI field amplitude is limited to the weaker of the two fields E1 and E2. From this we can get the electric field strength of each TI simulation.

Considering that the present work employs a dual-channel, four-electrode TI setup with 5 mA per electrode (i.e., 4 × 5 mA), we anticipated a corresponding increase in envelope amplitude compared with the 4 × 2 mA configuration used by Wessel et al. (3), who reported 0.22 V/m in the striatum. Scaling the single-electrode current from 2 mA to 5 mA provides a 2.5-fold increment, yielding approximately 0.55 V/m. Since the maximum TI amplitude can theoretically double under optimal conditions, the theoretical upper limit becomes approximately 1.1 V/m. To ensure that at least 50% of target voxels exceed this value after individual optimization, we set the inclusion criterion to 2.2 V/m (approximately 0.22 V/m × 10), providing a margin for variability and attenuation. This threshold was adopted as constraint (d) in the electrode placement screening protocol. Subsequently, through simulation studies of various electrode placement configurations and by applying the following method of exclusion, we identified the most suitable electrode placement scheme for each participant:

a) The ratio of the envelope electric field amplitude of the target brain region to the envelope electric field amplitude of the entire brain.

b) Maintain safe current densities, typically less than 2 mA/cm²

c) The magnitude of the envelope amplitude of the electric field in the target area is at least 2.2 V/m.

To identify the optimal four-electrode montage under the above constraints, a genetic algorithm (GA) was embedded within the individualized head-model and electric-field pipeline. The search space comprised the 81 positions of the extended 10–10 system; each chromosome encoded a unique, alphabetically sorted set of four electrode labels corresponding to the two stimulation channels. The fitness function invoked the previously validated TI solver to compute the peak electric field within a 10-mm-radius ROI centered at Montreal Neurological Institute (MNI) coordinates (28, 4, -4) in the striatum, while simultaneously enforcing the safety requirement that current density remain below 2 mA/cm²; non-compliant individuals received zero fitness. GA parameters were population size 50, crossover probability 0.8, mutation probability 0.1, elite retention 10, and termination after 200 generations or when the field strength reached 2.2 V/m. Selection combined roulette-wheel sampling with elitism; crossover employed single-point exchange followed by immediate deduplication and reordering; mutation replaced a single electrode at random. The algorithm returned the montage that maximized the focal electric field while satisfying all safety and selectivity criteria.

Through the above method screening, we obtained the optimal electrode placement position for each subject (Fig S1).


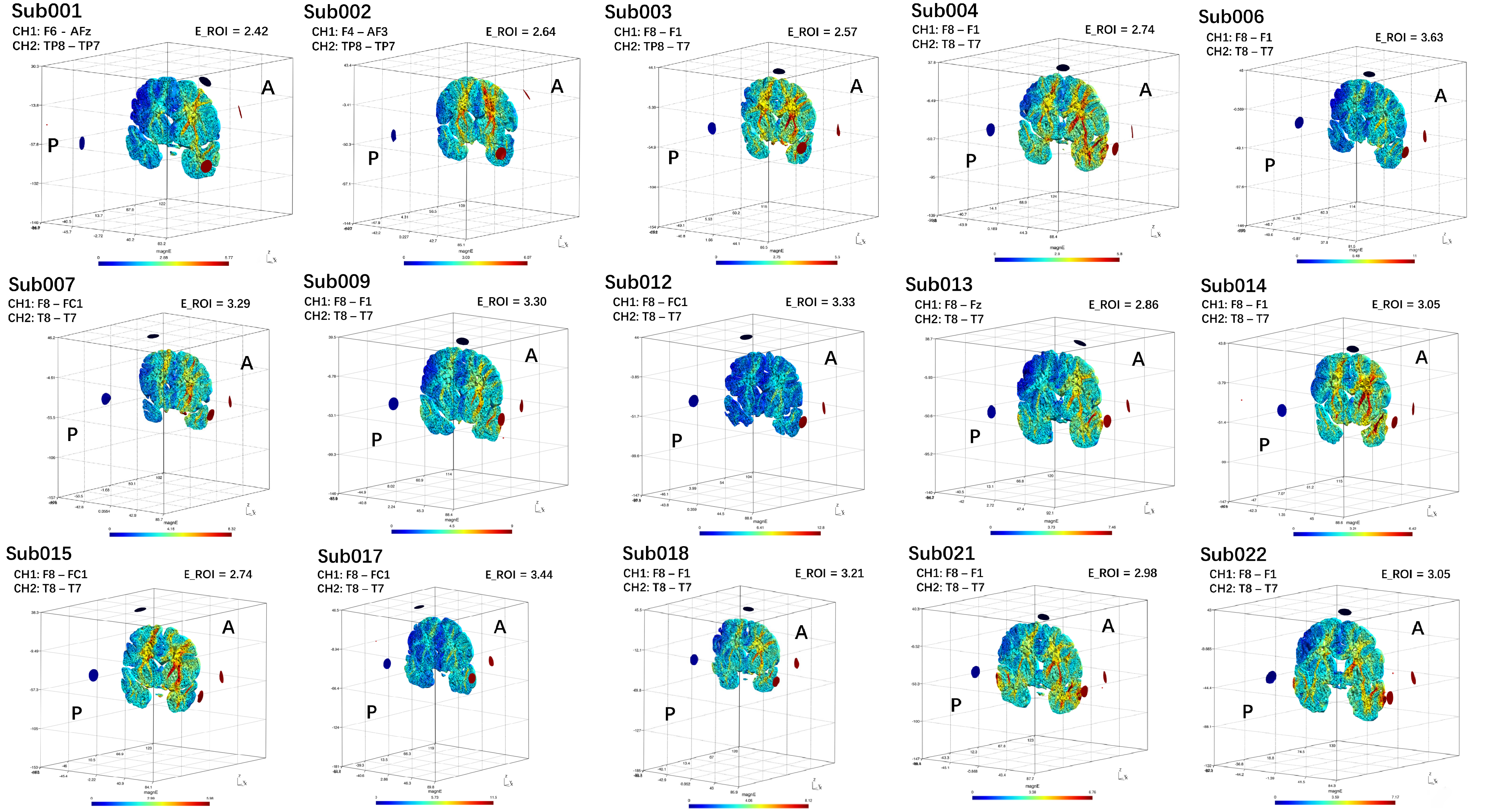


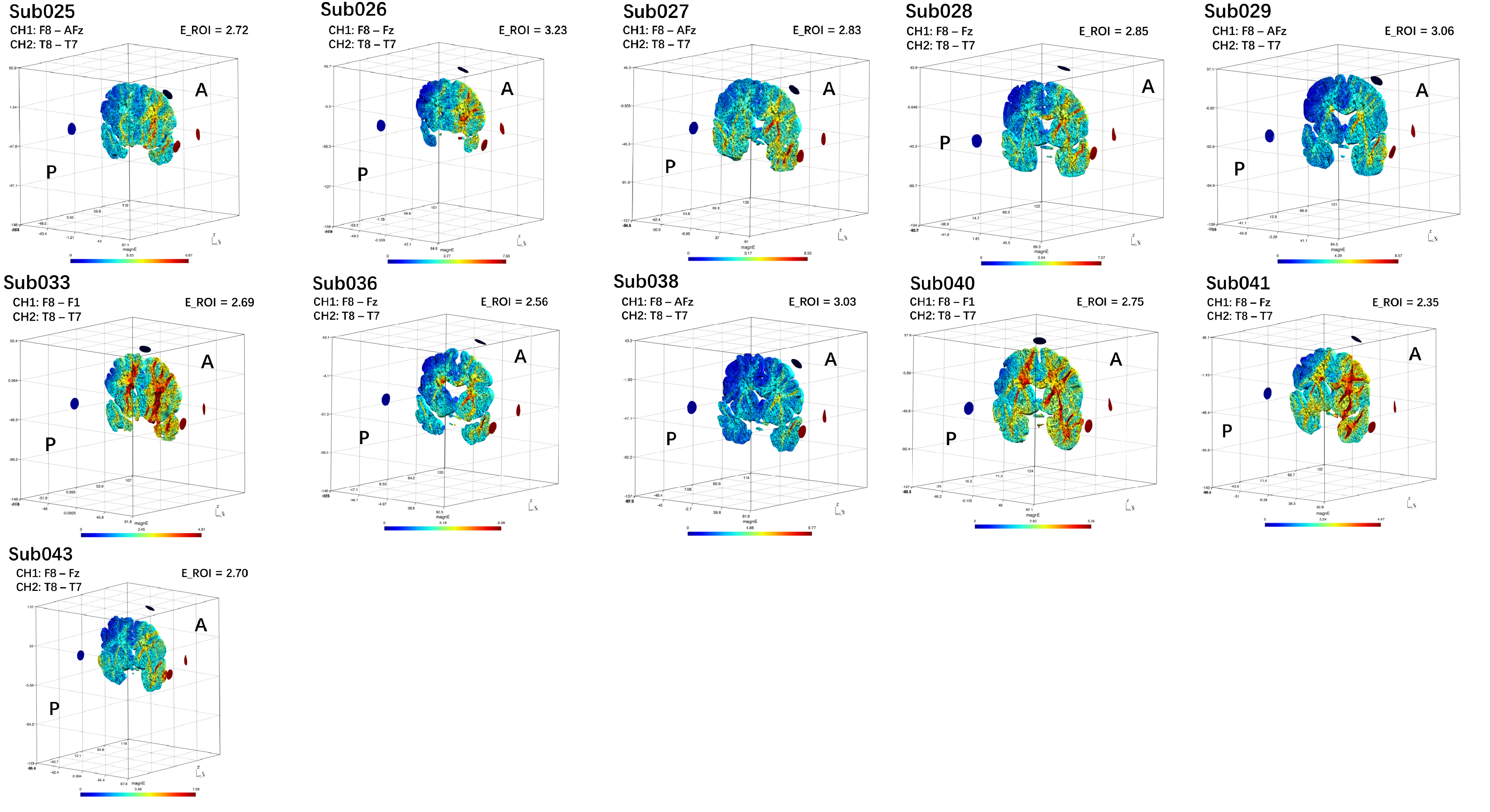


**Supplementary Figure 1.** Electric field intensity for participants. The modeling process included brain tissue segmentation, electrode placement (10-10 EEG system), finite element meshing, and TI electric field solution. A, Anterior; P, Posterior; E_ROI, Electric field intensity in the ROI area.

# Section B: Extended analysis of main results

**
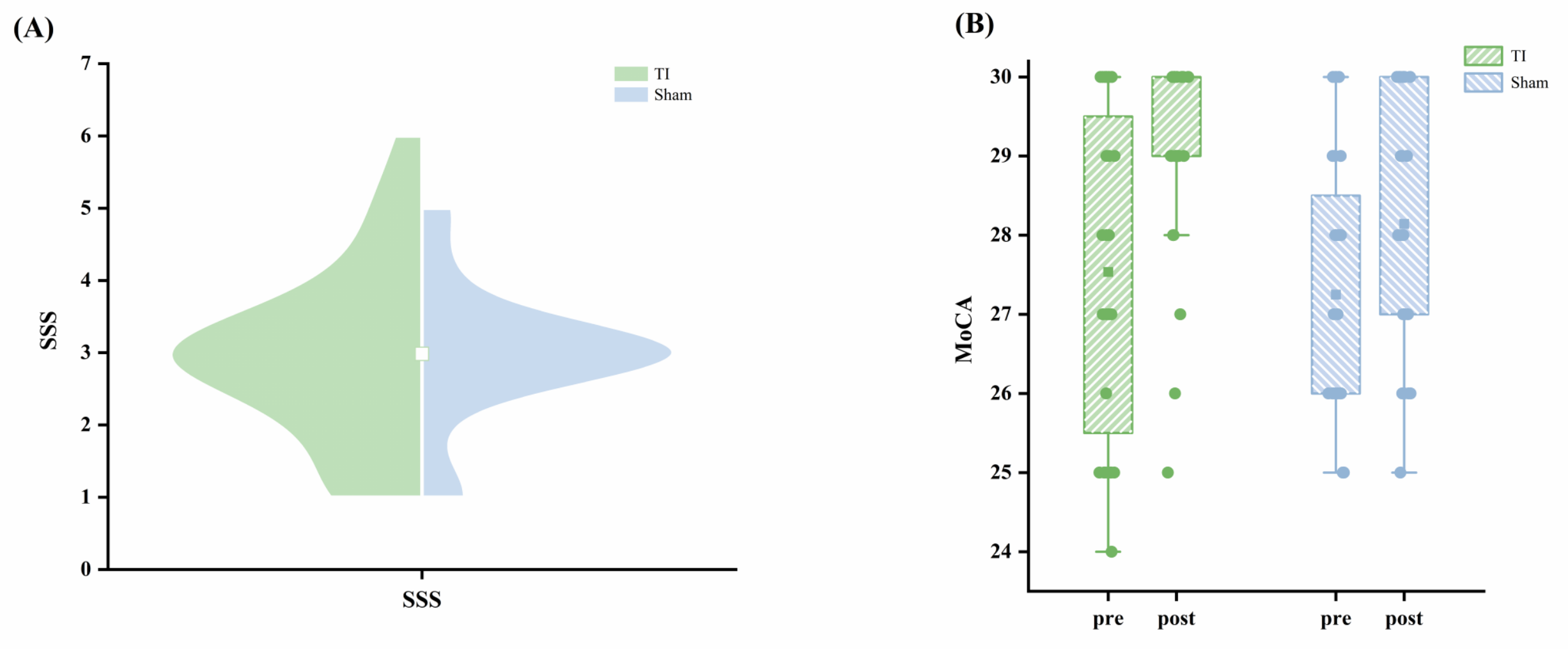
**

**Supplementary Figure 2.** Questionnaire responses about sleepiness and cognitive. Subjects were required to complete the questionnaire before and after the experiment to account for subject wakefulness and cognitive status (n=28). **(A)** Subjects’ level of attention and fatigue before the experiment, quantified with the Stanford Sleepiness Scale, ranging from 0 to 8. Comparison of pre-stimulation sleepiness in the TI and Sham groups. No significant difference was found (Pearson's chi-square test: χ^2^ = 4.275, P = 0.513). **(B)** Both groups of subjects completed the Montreal Cognitive Assessment Scale before and after the experiment. The results of the questionnaire data were compared for differences by Generalized Estimating Equations (GEE). The results showed that there was no significant difference between the two groups on the time x group interaction effect (Wald χ^2^ = 1.295, P = 0.255), and the group main effects analysis did not reveal differences (Wald χ^2^ = 2.655, P = 0.103). Time main effects analysis showed that post-experiment cognition was significantly higher than pre-experiment (Wald χ^2^ = 22.040, P < 0.001). TI, temporal interference stimulation; Sham, sham stimulation; Pre, before the experiment; Post, after the experiment; SSS, Stanford Sleepiness Scale; MoCA, Montreal Cognitive Assessment Scale. Boxes show median and interquartile range (IQR); whiskers extend to data within 1.5×IQR; points beyond are outliers.


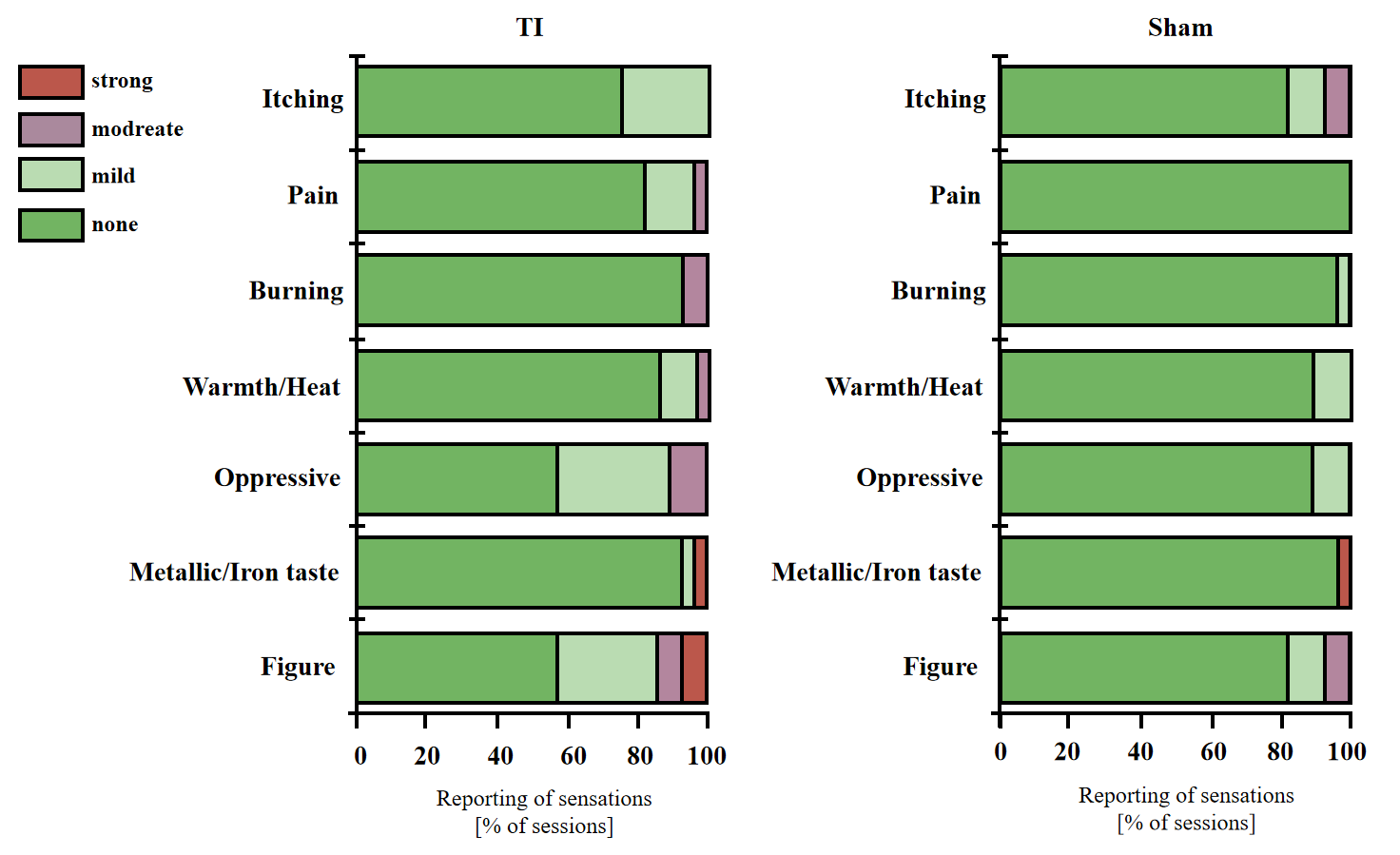


**Supplementary Figure 3.** Reported side effects of TI stimulation and sham stimulation. Subjects from both groups were asked to complete the Adverse Effects Questionnaires at the end of the experiment. The scale categorizes adverse reactions into 4 scales. The results of the questionnaire were analyzed using Pearson's chi-square test to determine the variability in the scores of the items between the two groups. The results showed that there was no significant difference between the two groups in terms of the total score of adverse reactions (χ2 = 10.864, P = 0.285). TI, temporal interference stimulation; Sham, sham stimulation.

**
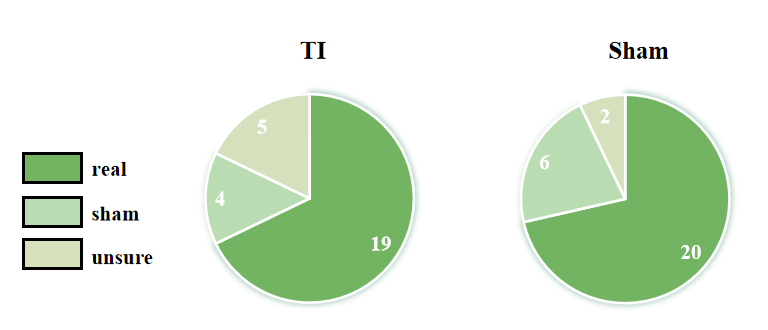
Supplementary Figure 4.** Blinding Efficacy of TI and Sham Stimulation. At the end of the experiment, subjects were asked to complete a blind school test questionnaire. The results of the questionnaire were tested by Pearson's chi-square and showed that without difference in the blinded test of stimuli across groups (χ2 = 1.711, P = 0.425).


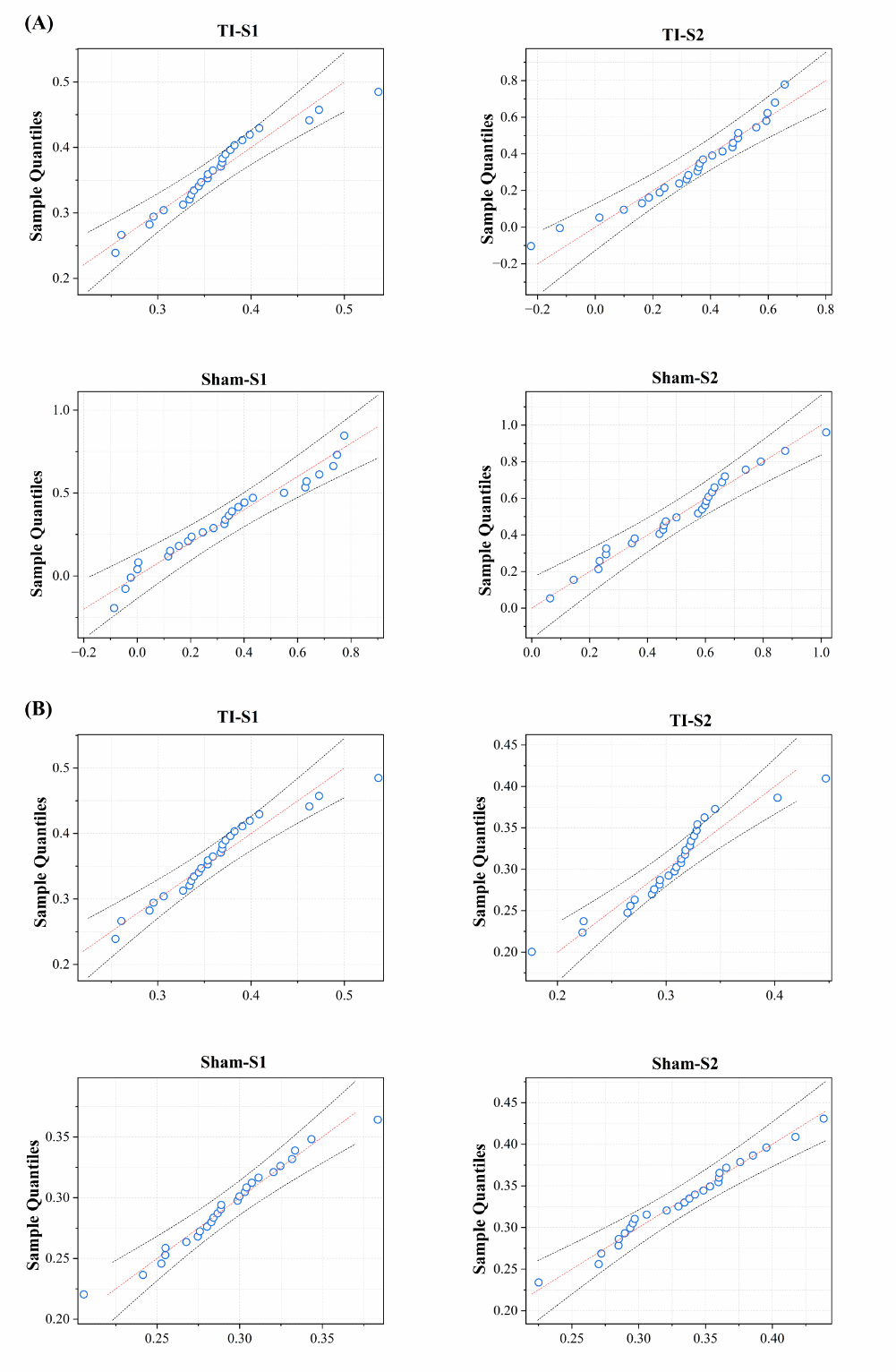


**Supplementary Figure 5.** QQ plots illustrating the normality of data across four groups. **(A)** Functional stability data. **(B)** Seed-based dFC variability data. Note. TI, temporal interference stimulation; Sham, sham stimulation; S1, denotes baseline; S2, during stimulation.

**Reference**

1. Thielscher A, Antunes A, Saturnino GB. Field modeling for transcranial magnetic stimulation: A useful tool to understand the physiological effects of TMS? Annu Int Conf IEEE Eng Med Biol Soc. 2015;2015:222-5.

2. Grossman N, Bono D, Dedic N, Kodandaramaiah SB, Rudenko A, Suk HJ, et al. Noninvasive Deep Brain Stimulation via Temporally Interfering Electric Fields. Cell. 2017;169(6):1029-41.e16.

3. Wessel MJ, Beanato E, Popa T, Windel F, Vassiliadis P, Menoud P, et al. Noninvasive theta-burst stimulation of the human striatum enhances striatal activity and motor skill learning. Nat Neurosci. 2023;26(11):2005-16.
